# Transforming Carbon Dioxide into Rocks!? Experiments for Understanding Carbon Dioxide Removal through Chemical Weathering

**DOI:** 10.1021/acs.jchemed.4c01276

**Published:** 2025-06-05

**Authors:** Philipp Spitzer

**Affiliations:** Center for Chemistry Education at the Institute of Chemistry, 27267 University of Graz, Heinrichstraße 28/VI, 8010 Graz, Austria

**Keywords:** Middle School Science, Introductory Science, Demonstrations, Environmental Chemistry, Hands-On Learning, Industrial Chemistry, Water Chemistry, Carbon Capture and Storage, Chemical Weathering

## Abstract

This paper presents a series of hands-on experiments designed to teach principles of carbon dioxide removal through chemical weathering in middle and high school chemistry classes. Chemical weathering is demonstrated by the reaction of carbon dioxide dissolved in water with basalt, forming bicarbonate. The experiments, which utilize simple materials such as PET bottles, are safe and feasible for classroom settings. Students are introduced to key concepts such as the carbon dioxide solubility in water and the chemical processes that trap carbon dioxide. The experimental design includes continuous monitoring of dissolved carbon dioxide levels using membrane-based carbon dioxide sensors and pH changes to visualize the progression of bicarbonate formation. These activities provide students with practical experience in carbon dioxide removal methods, mirroring real-world geological processes, and enhance their understanding of their part in the carbon cycle. In addition, the experiments also provide insight into fundamental processes of carbon capture and storage based on the chemical weathering of basalt. The accessibility of the materials, coupled with the relevance to current global climate discussions, makes this a valuable addition to chemistry education. This approach aims to fill the educational gap on carbon dioxide removal, empowering students to engage in societal discussions on the role of carbon capture and storage in the fight against climate change.

## Graphical abstract

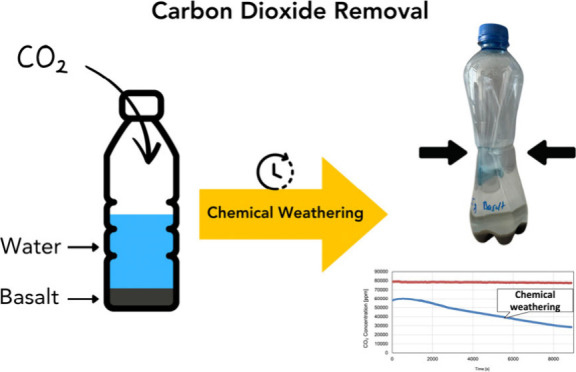

## Introduction

The carbon cycle is a fundamental Earth system process that regulates the exchange of carbon among the atmosphere, oceans, biosphere, and lithosphere. This cycle plays a crucial role in controlling atmospheric carbon dioxide levels, thereby influencing global climate patterns. In addition to biological driven storage, for example by plants, chemical weathering is an important mechanism for naturally storing carbon dioxide in nature and represents a natural negative feedback mechanism for Earth’s climate.^1^ As part of the carbonate-silicate cycle, chemical weathering regulates atmospheric carbon dioxide levels over geological time scales, with rates influenced by various environmental factors. For instance, rising temperatures accelerate chemical weathering, increasing carbon dioxide uptake and leading to long-term cooling by reducing atmospheric carbon dioxide concentrations.^2^ In contrast to biological storage, carbon dioxide can be sequestered for longer periods of time in the geochemical carbon cycle.

The process of chemical weathering can be mainly categorized into carbonate and silicate weathering, both of which contribute to natural carbon dioxide sinks, such as rivers in the Alps or glacial meltwater streams,^3^ which transport weathering products and further enhance carbon dioxide sequestration.^1,4,5^ A key contributor to silicate weathering is basalt, a widespread volcanic rock composed primarily of magnesium–iron-calcium silicates (pyroxenes), calcium- and sodium-rich feldspar (plagioclase), and olivine in variable composition.^6^ These minerals undergo chemical weathering when they react with carbonic acid, which forms through the dissolution of atmospheric carbon dioxide in water.^7^ To illustrate the process of silicate weathering, pyroxenes serve as an example. When exposed to carbonic acid, these silicate minerals undergo dissolution, leading to the release of metal cations into solution:^1,7,8^

1 $$(Mg,Fe,Ca)SiO_{3_{\left(s\right)}}+2CO_{2_{\left(g\right)}}+3H_{2}O_{\left(l\right)}⇌{\left(Mg^{2+},Fe^{2+},Ca^{2+}\right)}_{\left(aq\right)}+{2HCO_{3}^{-}}_{\left(aq\right)}+H_{4}SiO_{4_{\left(aq\right)}}$$

Once bicarbonate ions (HCO_3_^–^) are present in solution, further transformations can occur, depending on the pH and the concentration of calcium and magnesium ions. Carbonate minerals, which constitute a stable sink of carbon dioxideon geologic time scales, can precipitate at alkaline pH and sufficient ion concentrations and may also form from aqueous bicarbonate. While calcite (CaCO_3_) readily forms under ambient alkaline conditions, magnesium carbonates are known to form under specific natural conditions, such as during serpentinization processes at elevated temperatures and pressures.^9^

A key advantage of carbon dioxide sequestration through chemical weathering is the long-term stability of stored carbon. Unlike biological pathways such as reforestation, which are susceptible to carbon release through decomposition or wildfires, the products of silicate weathering—bicarbonate and carbonate minerals—represent a significantly more durable carbon sink. This distinction is crucial for understanding the role of different sequestration pathways in long-term climate mitigation.

The natural sequestration of atmospheric carbon dioxide through silicate weathering highlights the potential for leveraging this process in climate mitigation strategies. While these reactions occur over geological time scales, recent research has focused on accelerating chemical weathering as a means of Carbon Capture and Storage (CCS). Notable CCS projects have already demonstrated the feasibility of in situ carbon dioxide mineralization, where carbon dioxide is injected into basaltic formations, leading to rapid carbonate formation.^10,11^ Additionally, the application of rock powder to agricultural soils has been proposed as a scalable method for carbon sequestration, utilizing enhanced weathering to accelerate carbon dioxide drawdown.^12^

Although research on enhanced weathering and in situ mineralization has demonstrated the feasibility of using these processes for carbon sequestration, CCS alone is not a comprehensive solution to climate change. Instead, it is considered a complementary approach that may help mitigate a portion of residual carbon dioxide emissions, particularly from sectors where reductions are technologically challenging (e.g., cement production).^6,13,14^

Given the increasing scientific and political relevance of natural and industrial carbon sequestration, it is crucial that students gain a realistic and scientifically grounded understanding of these processes. However, carbonate-silicate weathering and CCS remain underrepresented in chemistry education despite their importance in climate discussions. Previous educational approaches have explored CCS through station-based learning,^15^ model experiments using activated carbon,^16^ and amine-based CO_2_ capture in PET bottle setups.^17^ Mauch and Rubner proposed experiments measuring carbon dioxide reduction using amines and bases, incorporating real-time carbon dioxide monitoring with an Arduino-based sensor.^18^ Some outreach initiatives of CCS-projects also engage students with practical CCS applications.^19^

A small, spontaneous survey of upper-level German chemistry and geography students (aged 17–19, N = 21) was conducted shortly before the legalization of CCS in Germany and showed that only three students were familiar with the concept, despite its presence in media discussions. Even though the number of students in the survey is small, one can assume that there is an educational gap. To address this, the following experiments have been designed to demonstrate key processes of chemical weathering.

## Rationale

To enable students to actively participate in current societal discussions on climate change, it is essential to bridge this knowledge gap by integrating chemical weathering into chemistry education. This work focuses on experimental methods designed to demonstrate and elucidate chemical weathering, particularly through its dissolution in water and subsequent chemical reactions with basalt to store carbon dioxide. In contrast to the industrial storage of carbon dioxide by the formation of carbonate, the experiments presented here only show storage by the formation of hydrogen carbonate due to the conditions at the school. However, this is also an important step in the formation of carbonate, and the process of industrial storage of carbon dioxide in basalt can be partially reproduced. Unlike conventional CCS techniques that involve storing carbon dioxide in deep rock formations, mining sites, or under the seabed—methods not feasible for classroom demonstrations—these experiments explore alternative storage mechanisms based on chemical weathering that are safe, practical, and easily implementable in a school setting.

## Experimental Overview

Videos of the experiments can be downloaded at the following link: https://glaciereducation.com/publications/transforming-carbon-dioxide-into-rocks/.

The following experiments demonstrate carbon dioxide sequestration through chemical weathering. The sequence begins by illustrating the solubility of carbon dioxide in water, followed by experiments that showcase the removal of carbon dioxide through its reaction with basalt. Each phenomenon is initially presented using simple demonstration experiments with PET bottles, making them accessible and practical in educational settings. The idea for this experimental setup using PET bottles originates from Asherman et al.,^17^ who demonstrated the reaction of carbon dioxide with sodium hydroxide as a possibility for CCS. This principle is adapted here to illustrate the removal of carbon dioxide through chemical weathering of basalt. To accurately measure dissolved carbon dioxide without affecting the solution, the membrane-based measurement method described by Johnson et al.^20^ is employed. This method allows carbon dioxide to diffuse through a gas-permeable membrane and be detected in a closed gas space, enabling the continuous monitoring of carbon dioxide levels. This membrane is suitable for popular carbon dioxide sensors produced mainly for schools and is therefore used in the following experiments.

### Demonstrating the Solubility of Carbon Dioxide in Water Using a PET-Bottle

The solubility of carbon dioxide in water is demonstrated through a simple experiment utilizing a PET bottle. To begin, 200 mL of distilled water is poured into a 0.5 L PET bottle, and a few drops of liquid universal indicator are added, which turn the water green, indicating a neutral pH. Carbon dioxide gas is introduced into the air space above the water; this can be safely accomplished using a carbon dioxide gas cylinder (e.g., one used with sodamakers). The bottle is immediately sealed tightly to prevent gas escape and is shaken vigorously. The dissolution of carbon dioxide into water causes a reduction in internal pressure, resulting in noticeable contraction of the PET bottle. The universal indicator shifts to yellow or orange, reflecting a decrease in pH due to the formation of carbonic acid (see Figure 1).

**Figure 1:**
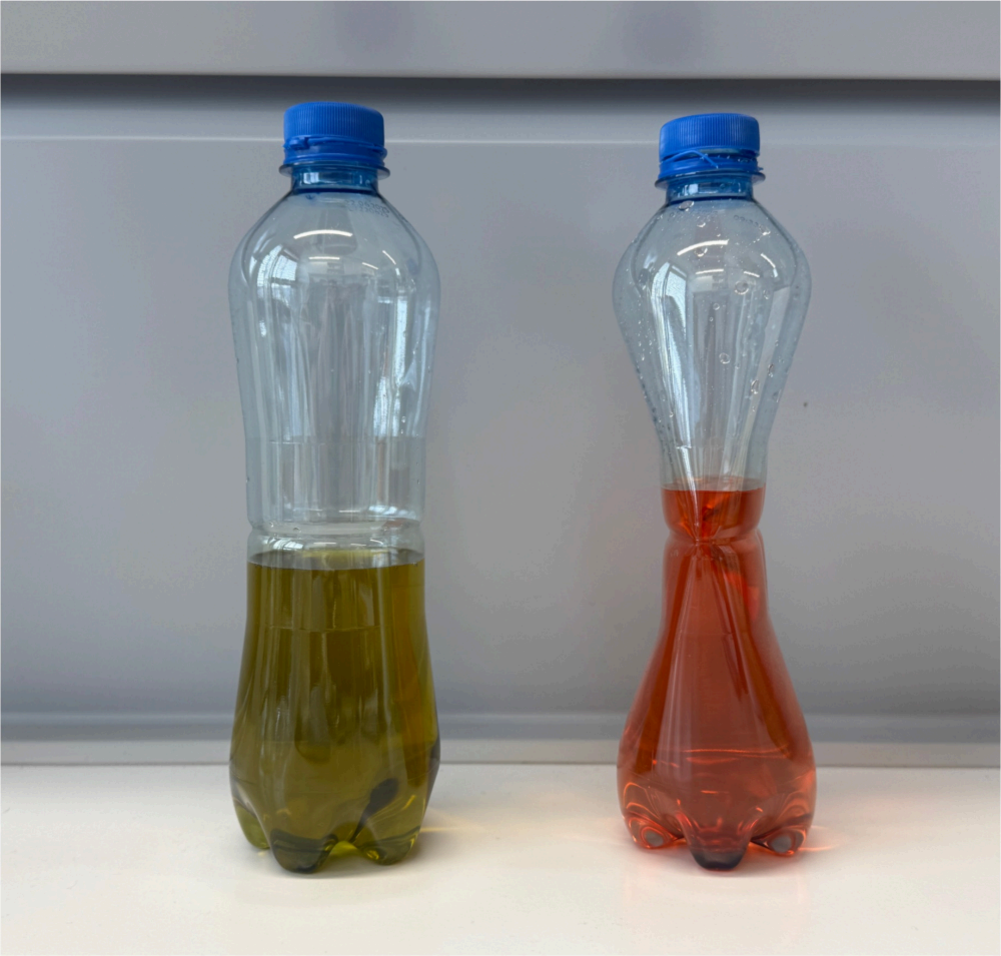
Change in pH value due to the dissolution of carbon dioxide in water. On the left, the bottle is shown before carbon dioxide was added, along with a sample mixed with a universal indicator (green). On the right, the bottle is shown after carbon dioxide has been added and dissolved in water, with the corresponding sample mixed with a universal indicator (orange or red).

By repeating the experiment with cold and warm water, it is demonstrated that more carbon dioxide dissolves in cold water, which can be shown to be reproducibly align with the temperature dependence of gas solubility. This confirms that carbon dioxide solubility increases with decreasing temperature, consistent with Henry’s law, which states that gas solubility in a liquid is inversely proportional to temperature.

These results not only demonstrate the temperature dependence of carbon dioxide solubility but also reinforce the concept of glacial rivers acting as effective natural carbon dioxide sinks due to their cold temperatures enhancing carbon dioxide absorption from the atmosphere.

### Demonstrating Chemical Weathering Traps Carbon Dioxide Using a PET-Bottle

Building upon the concept of carbon dioxide solubility, the next experiment provides insight into how dissolved carbon dioxide can be chemically converted and sequestered through reactions with basalt—a process central to chemical weathering. This hands-on experiment adapts the PET bottle setup to demonstrate the reaction between carbon-dioxide-enriched water and basalt powder. First, carbonated water is prepared by filling a 0.5 L PET bottle with 200 mL of distilled water. Carbon dioxide is introduced into the bottle, which is then sealed tightly and shaken vigorously. This process is repeated multiple times until the bottle no longer contracts upon shaking, indicating that the water is saturated with carbon dioxide. In a separate PET bottle, 20 g of finely ground basalt powder (particle size 0–0.2 mm) are placed. The carbonated water is carefully poured into the bottle containing the basalt powder. The bottle is sealed securely and shaken thoroughly to ensure that the basalt is well suspended in the carbon dioxide enriched water. Over the course of several days, the bottle is observed for physical changes. After 1 day, the bottle should become noticeably easier to compress, and by the second day, a visible change in the bottle’s shape will be evident (see Figure 2).

**Figure 2:**
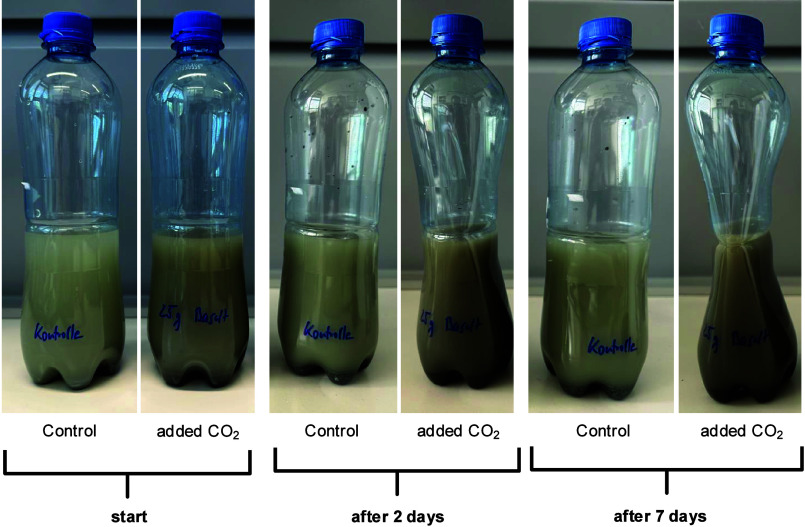
Basalt with water and carbon dioxide over time. On the left, you can see a control sample with basalt and water without the addition of carbon dioxide. The image clearly shows the dented bottle after a few days.

This softening of the bottle indicates a reduction in internal pressure due to the consumption of carbon dioxide, as it reacts with the basalt to form bicarbonate dissolved in water. The water can be decanted. If sodium hydroxide is added, and the pH is raised, carbonate will precipitate.

### Measuring Trapping of Dissolved Carbon Dioxide by Chemical Weathering

To quantitatively demonstrate carbon dioxide capture via chemical weathering, the decrease in the dissolved carbon dioxide concentration during the reaction with basalt is measured. A suspension of basalt powder (particle size 0–0.2 mm) in distilled water is prepared within an airtight reaction vessel equipped with a stirring mechanism and a carbon-dioxide sensor that utilizes a gas-permeable membrane.

Carbon dioxide is introduced into the suspension by carefully bubbling it through the mixture until a measurable concentration is achieved, ensuring the sensor’s operating range is not exceeded. The vessel is immediately sealed with the carbon-dioxide sensor in place to prevent gas escape (see Figure 3). Continuous stirring ensures even distribution of basalt particles and dissolved carbon dioxide, simulating natural conditions of chemical weathering.

**Figure 3:**
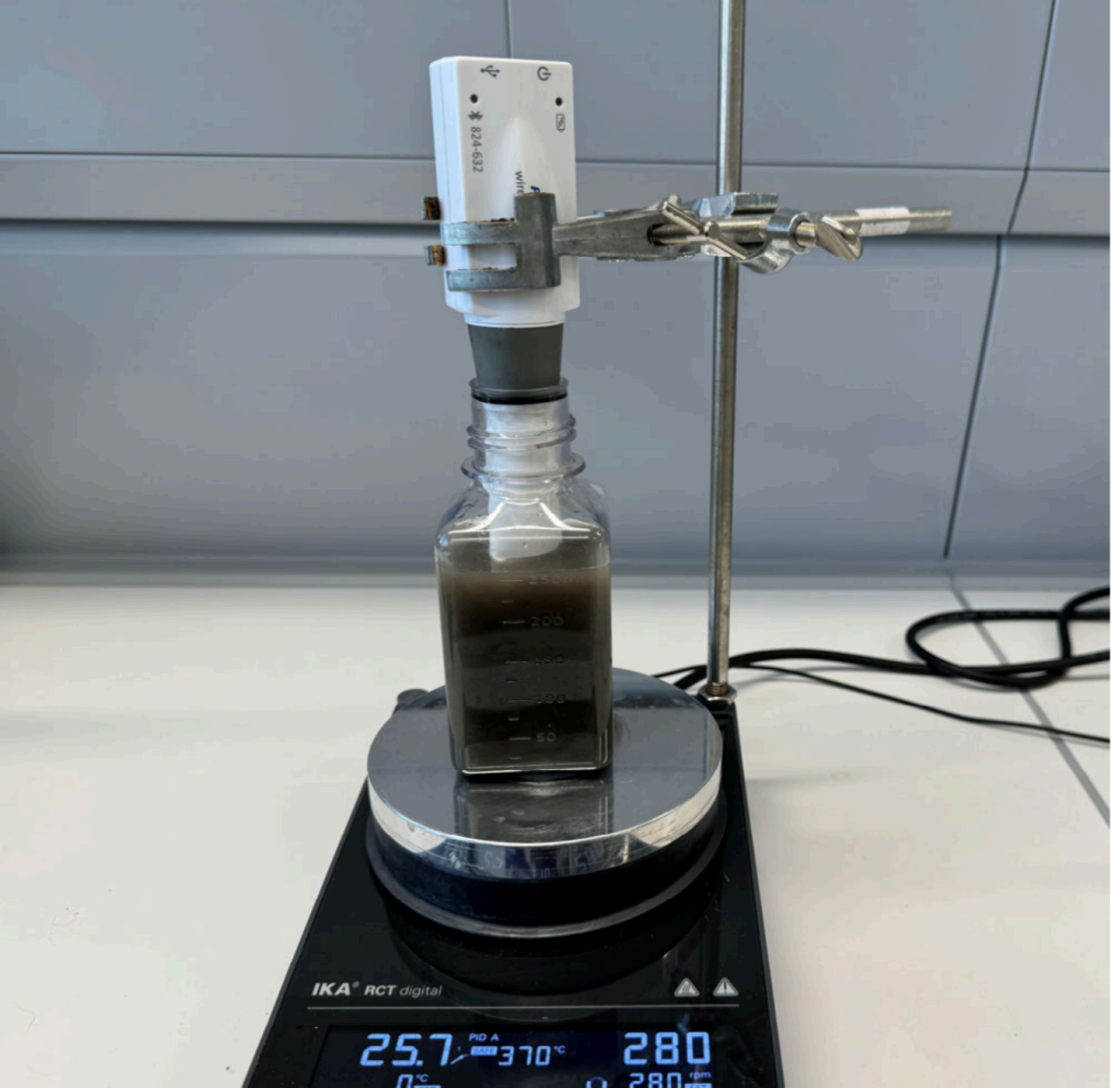
Experimental setup: Bottle filled with basalt powder and carbon dioxide enriched distilled water with a CO_2_-sensor.

The dissolved concentration of carbon dioxide is monitored over time. Figure 4 presents the carbon dioxide dissolved in water recorded over a period of 2.5 h. The blue curve represents the experiment with basalt powder, showing a significant decrease in the dissolved concentration of carbon dioxide. The orange curve is a control experiment conducted under identical conditions but without basalt powder, showing a relatively constant level. The comparison indicates that the presence of basalt facilitates the consumption of dissolved carbon dioxide through chemical reactions forming bicarbonate.

**Figure 4:**
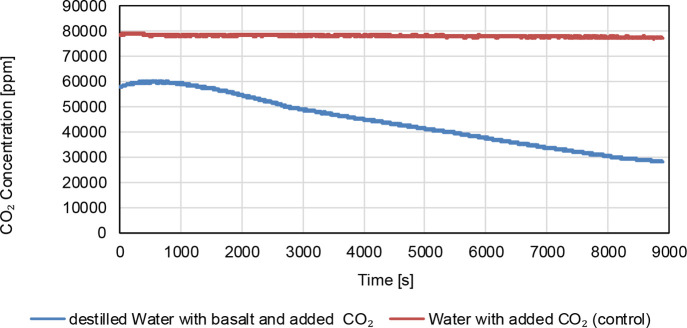
Measurement (2.5 h) of the carbon dioxide concentration in the gastight sealed bottle. The measurement with basalt powder is shown in blue and a control measurement without basalt powder is shown in orange.

The gastight nature of the vessel is confirmed by the constant level of carbon dioxide in the control experiment, ruling outgas leakage as a cause for the observed decrease. This quantitative measurement confirms the effectiveness of basalt in carbon dioxide removal through chemical weathering, providing empirical evidence of the process.

### Measurement of Rising pH Value Due to Bicarbonate Formation in the Process of Chemical Weathering

The progress of the chemical weathering reaction is also monitored by measuring the pH of the solution over time. As carbon dioxide is consumed and bicarbonate is formed, the acidity of the solution decreases, leading to a rise in pH. To measure this change, the reaction is set up in a vessel that can be sealed airtight with the pH-Sensor to prevent gas exchange with the environment.

In the experimental setup, a suspension of basalt powder in carbon-dioxide-saturated distilled water is prepared, like in the previous experiment. A pH probe is inserted into the solution, and the vessel is sealed around the probe to maintain a closed system. Continuous stirring is important to keep the basalt particles evenly distributed.

Figure 5 illustrates the results of a long-term pH measurement over approximately 25 h. The data show a gradual increase in pH from acidic toward neutral and eventually into the alkaline range. The increase in alkalinity is consistent with the formation of bicarbonate.

**Figure 5:**
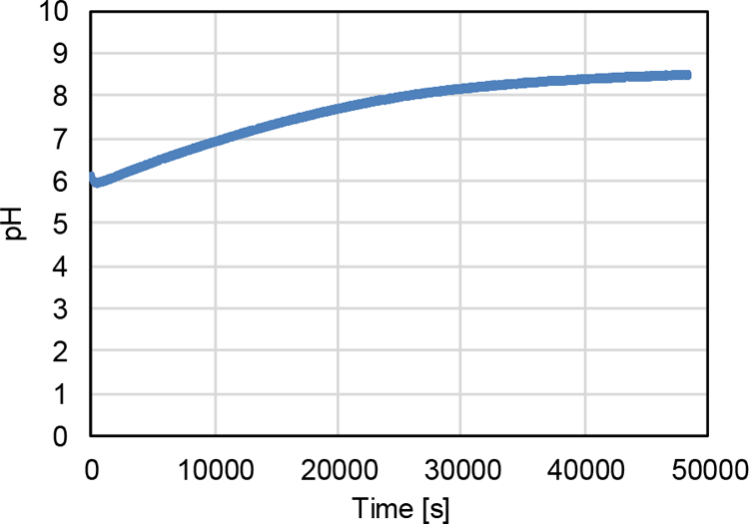
Long-term measurement (25 h) of the pH value. An increase in the pH value into the alkaline range can be observed.

Monitoring pH changes provides a straightforward and effective method to visualize and quantify the sequestration of carbon dioxide through chemical weathering, reinforcing the concepts demonstrated in previous experiments.

### Rock Dust as a Cheap Substitute for Basalt Powder

While the experiments described thus far utilize basalt powder, which may need to be sourced from specialized suppliers, alternative materials can be used to achieve similar results. Volcanic rock dusts, such as "glacial rock dust", "volcanic rock dust", or "diabase", are commercially available as soil amendments in garden centers and agricultural supply stores. These products typically consist of finely ground volcanic rocks rich in minerals similar to basalt and are particularly susceptible to chemical weathering processes. Using rock dust as a substitute offers a cost-effective and accessible option for conducting the experiments.

## Hazards

The basalt powder used in this instance did not have a GHS label. However, it is important to note that the dust from mafic minerals, i.e., minerals that contain a lot of magnesium and iron, should not be inhaled. Therefore, wearing an N95 mask (or better) or working in a fume hood is advised during weighing. It is also recommended to wear the protective equipment normally used in the laboratory (e.g., laboratory goggles). These lower risks are an advantage of the series of reported experiments compared with such model experiments using amines or alkalis.

## Results and Discussion

The experiments effectively demonstrate chemical weathering and illustrate the function of natural nonbiological carbon dioxide sinks. The initial solubility tests confirmed that carbon dioxide is more soluble in cold water than in warm water, which illustrates the solubility of carbon dioxide and the formation of carbonic acid. It can also help to understand mountain streams and glacier rivers as natural carbon dioxide sinks and underscores the significant role glacial rivers play in sequestering atmospheric carbon dioxide.

Subsequent experiments showed that dissolved carbon dioxide reacts with basalt to form bicarbonate, thereby reducing the amount of dissolved carbon dioxide and increasing the solution’s pH-value. Measurement data indicated a significant decrease in dissolved carbon dioxide when basalt was present, while control experiments without basalt or carbon dioxide showed a minimal change, confirming the effectiveness of basalt in the sequestration of carbon dioxide. This reaction mirrors natural chemical weathering processes, where minerals react with carbonic acid to form solid carbonates. Unfortunately, due to the kinetics of basalt dissolution and carbonate precipitation, only the conversion of dissolved carbon dioxide to aqueous bicarbonate can be observed. This was demonstrated by additional measurements using an X-ray diffractometer. The lack of carbonate formation in this experiment is likely due to a combination of factors such as low ion concentrations and the absence of conditions required for reaching supersaturation with respect to carbonate minerals.

Although the reaction times of the experiments presented here are longer compared to amine-based methods—requiring several hours to days—the experiments presented here can be integrated into lesson plans through careful scheduling. For instance, initiating the experiments during one class period and analyzing the results in subsequent sessions will allow students to observe the progression of the reactions. Due to their extended duration, the experiments were presented here as demonstration experiments. However, they can also be carried out by students.

The reported measurements can only be regarded as semiquantitative. Since it was important that the experiments were carried out easily in school, the addition of carbon dioxide in the experiments was not standardized. The use of dry ice as a source of carbon dioxide could possibly contribute to standardization here. However, the implementation of precise measurements is not planned here. Rather, the aim is to generally address the concept of chemical weathering in school and to use experiments to demonstrate which current options are currently being discussed in research on CCS.

The simplicity, safety, and environmental relevance of these experiments make them highly suitable for classroom settings, enhancing student understanding of chemical weathering and the importance of these natural processes in the carbon cycle.

## Conclusion and Outlook

These experiments demonstrate the role of chemical weathering in carbon dioxide sequestration in nonbiological carbon sinks. By demonstrating the solubility of carbon dioxide in cold water and its subsequent reaction with basalt to form bicarbonate, they provide a practical and educational approach to understanding natural carbon sinks. This approach mimics real-world processes, for example, found in glacial rivers. The use of simple materials like PET bottles and readily available basalt powder or alternatives makes these experiments accessible for educational settings, enabling students to visualize and measure carbon capture in real-time. While carbonate precipitation was not observed under experimental conditions, bicarbonate formation remains a crucial intermediate step in carbon dioxide sequestration as used for CCS.

## Supplementary Material





## Notes

Instructor notes, materials and costs (PDF, DOCX)
