# Supplementary material for: Transforming Carbon Dioxide into Rocks!? Experiments for Understanding Carbon Dioxide Removal through Chemical Weathering
Source: J Chem Educ. 2025 Jun 5;102(7):3065–70. doi: 10.1021/acs.jchemed.4c01276 (PMC12244487; doi:10.1021/acs.jchemed.4c01276)
Supplement: Supplementary file 1 [file ed4c01276_si_001.pdf]

Supporting Information - Instructors

Transforming Carbon Dioxide into rocks!? - Experiments for understanding Carbon Dioxide Removal through Chemical Weathering

Philipp Spitzer*

Center for Chemistry Education at the Institute of Chemistry, University of Graz, Heinrichstraße 28/VI, 8010 Graz, Austria

*Corresponding Author E-Mail: philipp.spitzer@uni-graz.at

MATERIALS AND COSTS

Following basic laboratory equipment is required for the experiments:

- Magnetic stirrer
- Stirring bar
- Scales
- Distilled water
- universal indicator

In the following Table S1 is an overview of all additionally used materials and sensors.

Table S1. Additionally used materials and sensors

MATERIAL AND CHEMICALS	COMMENT	APPROXIMATE COSTS
0.5L PET-Botte		0 €
Basalt powder (grain size 0-0.2mm)	Instead you can use “rock dust”, “vulcanic rock”, or "diabase," which commercially available as soil amendments in garden centers and agricultural supply stores	6 €/kg
CO_2 dispenser	In our own experiment, a CO_2 corkscrew was used. It is also possible to use a sodachargers or a gas bottle.	
Wireless CO_2-Sensor	For this article we used the Pasco®-sensor for the semi-quantitative measurement of dissolved CO_2 because of their Waterproof Sleeve. Sensors from other companies can also be used.	250 €
Pasco® Dissolved CO_2 Waterproof Sleeve		30 €
pH-Sensor	If you also want to measure the change in pH value, a pH sensor is required.	

EXPERIMENTAL INSTRUCTIONS

The experiments described in the article are detailed below, along with helpful commentary. The order of the experiments follows the structure outlined in the article. Each section begins with a demonstration experiment using the PET bottle, followed by a semi-quantitative measurement with a sensor.

Demonstrating the solubility of CO_2 in water using a PET-Bottle

To conduct these experiments, you'll need an empty 0.5-liter PET bottle, distilled water, a source of CO_2-gas (e.g. a soda charger). Begin by filling the bottle one-third with distilled water. Release some CO_2-gas into the air above the water. Shake the bottle gently to ensure thorough mixing and the dissolution of carbon dioxide into the water. This setup provides a simple but impressive experiment for observing CO_2 absorption in water.

To demonstrate the different solubility of carbon dioxide in warm and cold water, the experiment can be carried out with warm and cold water. While almost no gas dissolves in the warm water after the second run, the experiment can be carried out several times with the cold water.

With the help of a liquid universal indicator, the acidification of the water through the formation of carbonic acid can also be shown.

Demonstrating Chemical weathering traps carbon dioxide using a PET-bottle

Analogous to the demonstration experiment on the solubility of carbon dioxide in water, the process of carbon capture and storage (CCS) via chemical weathering can also be demonstrated. To begin, fill a 0.5L PET bottle with 25g of basalt powder, then add distilled water until the bottle is half full. Unlike the previous experiment, carbon dioxide is now introduced directly into the distilled water, and the bottle is gently swirled.

Some of the carbon dioxide will dissolve in the water, so it's important to introduce a sufficient amount of gas. However, a CO_2-enriched atmosphere will remain inside the bottle. No noticeable changes should occur when the bottle is shaken. If the bottle contracts during shaking, reopen it and introduce more carbon dioxide. Once enough gas has been added, close the bottle and observe it over the next few days. After two days, a slight change should be visible, and after a week, it will be clear that the bottle has contracted (see figure 1).

Instead of basalt powder, so-called “primary rock flour” or “rock dust” can also be used. This is sold as a natural fertilizer. It consists largely of diabase and thus also of volcanic rock such as basalt.

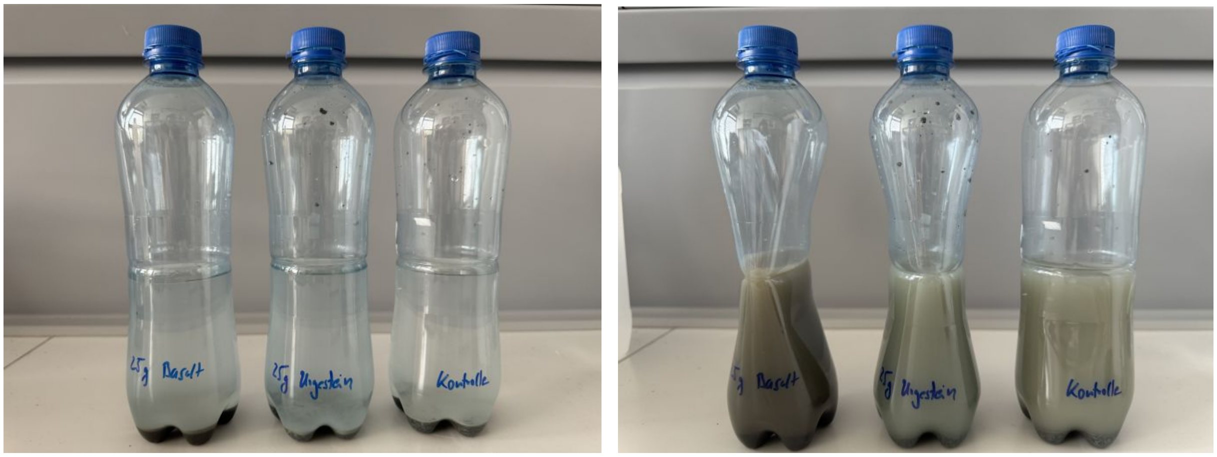

Figure 1: On the left you can see the filled PET bottles (from left to right: basalt powder, rock dust, control without CO2). The right picture shows the bottles after a few days.

Measuring trapping of dissolved carbon dioxide by chemical weathering

The previous experiment demonstrated the binding of carbon dioxide through chemical weathering. This process can also be observed quantitatively using a CO_2 sensor. To do this, add 10g of basalt powder to the reaction bottle that typically comes with the CO_2 sensor (these bottles are usually sealed gas-tight by the sensor). Next, pour 250mL of distilled water into the container and place a stir bar inside. Introduce carbon dioxide into the container, ensuring the sensor—covered by a gas-permeable but waterproof membrane—is immersed in the water. The sensor or membrane should be mostly submerged. Seal the bottle hermetically with the sensor.

Before starting the measurement, wait 1–2 minutes to allow the micro-atmosphere between the membrane and sensor to stabilize. Then, activate the stir bar on a magnetic stirrer, setting it to around 280 rpm, and begin the measurement. For faster results, add just enough carbon dioxide to avoid exceeding the sensor’s maximum limit. Even if the concentration of carbon dioxide initially exceeds the sensor’s range, the measurement can still proceed, though it will take a bit longer to stabilize.

Measurement of rising pH value due to carbonate formation in the process of chemical weathering

In previous experiments, a decrease in dissolved carbon dioxide was observed. To provide evidence of the chemical reaction and the formation of carbonate, the pH value can be measured as an indicator throughout the reaction. This can be done alongside the CO_2 measurement from the previous experiment by using the same reaction vessel, or in a separate experiment with the same setup. The amounts of distilled water and basalt powder should remain consistent with the previous experiment.

When measuring the pH separately, it's important to note that the results will be semi-quantitative, as the amount of carbon dioxide introduced may vary between experiments.

DEMONSTRATION VIDEOS

Videos of the demonstration of the solubility of carbon dioxide in distilled water using a PET bottle as well as the experimental preparation for the start of the CCS measurement series by Chemical Weathering can be accessed at the following link:

https://glaciereducation.com/publications/transforming-carbon-dioxide-into-rocks/
